# H2A.Z-Mediated Localization of Genes at the Nuclear Periphery Confers Epigenetic Memory of Previous Transcriptional State 

**DOI:** 10.1371/journal.pbio.0050081

**Published:** 2007-03-20

**Authors:** Donna Garvey Brickner, Ivelisse Cajigas, Yvonne Fondufe-Mittendorf, Sara Ahmed, Pei-Chih Lee, Jonathan Widom, Jason H Brickner

**Affiliations:** Department of Biochemistry, Molecular Biology, and Cell Biology, Northwestern University, Evanston, Illinois, United States of America; National Cancer Institute, United States of America

## Abstract

Many genes are recruited to the nuclear periphery upon transcriptional activation. The mechanism and functional significance of this recruitment is unclear. We find that recruitment of the yeast *INO1* and *GAL1* genes to the nuclear periphery is rapid and independent of transcription. Surprisingly, these genes remain at the periphery for generations after they are repressed. Localization at the nuclear periphery serves as a form of memory of recent transcriptional activation, promoting reactivation. Previously expressed *GAL1* at the nuclear periphery is activated much more rapidly than long-term repressed *GAL1* in the nucleoplasm, even after six generations of repression. Localization of *INO1* at the nuclear periphery is necessary and sufficient to promote more rapid activation. This form of transcriptional memory is chromatin based; the histone variant H2A.Z is incorporated into nucleosomes within the recently repressed *INO1* promoter and is specifically required for rapid reactivation of both *INO1* and *GAL1*. Furthermore, H2A.Z is required to retain *INO1* at the nuclear periphery after repression. Therefore, H2A.Z-mediated localization of recently repressed genes at the nuclear periphery represents an epigenetic state that confers memory of transcriptional activation and promotes reactivation.

## Author Summary

Eukaryotic cells control the spatial arrangement of chromosomes; the localization of genes can both reflect and contribute to their transcriptional state. A number of genes in the simple eukaryote brewer's yeast are “recruited” to the nuclear periphery through interactions with the nuclear pore complex when they are expressed. The functional significance of peripheral recruitment is unclear.

Here, we show that recruited genes are actively retained at the periphery for generations after transcription is repressed. This suggests that localization at the nuclear periphery represents a novel inherited state that might allow simple eukaryotic organisms to “remember” previous transcriptional activation. This type of memory allows for more robust reactivation of genes, suggesting that it is adaptive. Finally, both retention at the nuclear periphery and rapid reactivation require a variant form of histone H2A.

Adaptive memory is distinct from other types of transcriptional memory. In developmental memory, transcriptional states established by transcriptional regulators early in embryogenesis are propagated long after these regulators have disappeared. Adaptive memory does not propagate a state, but represents a novel state that serves as a source of information. In this way, it resembles a rudimentary form of cellular learning that allows cells to benefit from recent experience.

## Abstract 3

Recruitment of active genes to the periphery of the yeast nucleus does not require concurrent transcription.

## Introduction

The subnuclear localization of DNA has important roles in regulating transcription [1,2]. In particular, localization of chromatin near the nuclear periphery has well-documented effects on transcription. Heterochromatin and developmentally repressed genes localize at the nuclear periphery in metazoan cells, and peripheral localization promotes silencing of telomeres and the mating type loci in yeast [1,3–5]. Conversely, recent studies have shown that certain genes are conditionally recruited to the nuclear periphery when transcriptionally activated in both yeast and mice [6–13]. The yeast genes *INO1* and *GAL1* distribute randomly within the nucleoplasm under repressing conditions, but become co-localized with the nuclear periphery upon activation [6,7]. Live-cell four-dimensional imaging experiments reveal that recruitment is associated with both a change in the subnuclear distribution of genes and a reduction in their mobility, resulting in constrained movement near the nuclear envelope [9,14]. Chromatin immunoprecipitation experiments suggest that these and many other transcriptionally active genes physically interact with components of the nuclear pore complex (NPC) and associated factors [7].

The mechanism and functional significance of peripheral localization is unclear. Interaction of *GAL1* with the nucleoporin Nup2 requires the Gal4 activator, but does not require the SAGA histone acetylase complex, and is not affected by inactivation of RNA polymerase II [13]. Thus, the association with the NPC and, presumably, recruitment of these genes to the nuclear periphery are regulated upstream of TBP binding and transcription. Furthermore, artificial tethering at the nuclear periphery promotes transcriptional activation of the *INO1* gene [6] and the *HXK1* gene [8], and is sufficient to activate certain reporter genes [11]. Thus, recruitment to the nuclear periphery appears to have a functional role in promoting transcriptional activation.

In contrast, recruitment of genes to the nuclear periphery has also been suggested to reflect coupling between transcription and mRNA export. Chromatin immunoprecipitation studies suggest that the interaction of mating pheromone–induced genes with the NPC is mediated by the mRNA [12]. Likewise, recruitment of *HXK1* and *GAL1* to the nuclear periphery is affected by sequences in the 3′ UTR [8,15], and recruitment of *GAL1* requires proteins that have been implicated in mRNA export [9]. These results raise the possibility that recruitment of genes to the nuclear periphery might simply be the product of physical interactions between nascent transcripts, the mRNA export machinery, and the NPC.

Using a quantitative chromatin localization assay [6], we find that the transcriptional activation of both *INO1* and *GAL1* genes in yeast is biphasic, with the mRNA levels increasing dramatically after gene recruitment is complete. RNA polymerase II activity was not required for peripheral recruitment of *INO1*. Furthermore, when cells were shifted from activating to repressing conditions, *INO1* and *GAL1* remained localized at the nuclear periphery for generations. We find that localization at the periphery defines a distinct, heritable state that marks recently repressed genes and promotes reactivation. The reactivation of *GAL1* was more rapid in cells that had previously activated the gene, even after six generations of repression. The rate of activation of *INO1* was accelerated when the gene was artificially tethered to the nuclear envelope and was delayed in a mutant blocked for gene recruitment.

Epigenetic mechanisms of transcriptional memory are employed extensively during metazoan development to stably propagate transcriptional states [16]. Such memory can be mediated by DNA methylation [17], by histone H3 acetylation and methylation [18,19] or by incorporation of variant histone H3.3 [20]. We find that the histone variant H2A.Z was specifically required for reactivation of recently repressed *INO1* and *GAL1,* but had no role in the activation of the long-term repressed states of these genes. H2A.Z associated with nucleosomes in the promoter of the recently repressed *INO1* gene, but not in the promoter of either activated or long-term repressed *INO1*. Finally, we find that H2A.Z is essential for retention of recently repressed *INO1* at the nuclear periphery. These results identify a new form of chromatin-based transcriptional memory and highlight an important role for H2A.Z in regulating subnuclear localization to mark recently repressed genes and promote their reactivation.

## Results

### Rapid Gene Recruitment to the Nuclear Periphery upon Transcriptional Activation

**Figure 1:**
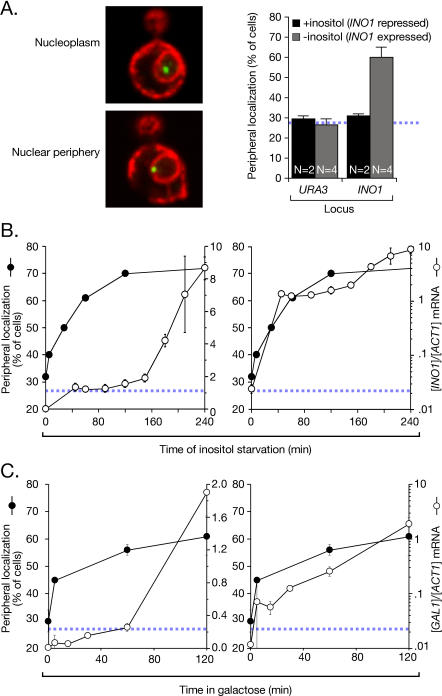
Recruitment of *INO1* and *GAL1* to the Nuclear Periphery Is Rapid (A) Left: merged confocal micrographs of cells stained for Lac I-GFP (green) and Sec63-myc (red), and scored as peripheral or nucleoplasmic. Right: cells having the lac repressor array integrated either at *URA3* (strain JBY409) or *INO1* (JBY397) were grown in the presence or absence of inositol, and scored for peripheral localization as described [6]. Data are averages of multiple replicates (indicated as N) from independent cultures. Each replicate represents 30–50 cells. The hatched blue line represents the baseline level of peripheral localization for the *URA3* gene. (B) At the indicated times after removal of inositol, cells were scored for peripheral localization of *INO1* (filled circles, extrapolated to 300 min [Figure S1]; two replicates of 30–50 cells). Also, *INO1* mRNA abundance was quantified using RT Q-PCR and expressed relative to *ACT1* mRNA (open circles; [58]). Left panel: both datasets plotted on a linear scale. Right panel: the mRNA abundance was plotted on a logarithmic scale, and the localization was plotted on a linear scale. (C) The localization of the *GAL1* gene (two replicates of 30–50 cells) and the *GAL1* mRNA abundance were quantified in strain DBY32 and plotted as in (B).

To determine whether gene recruitment to the nuclear periphery requires transcription, we used a chromatin localization assay [6]. This is a quantitative assay for localization of genes at the nuclear periphery based on a system developed by Belmont, Murray, and colleagues [21,22]. An array of 128 lac repressor–binding sites is targeted for integration to a location in the yeast genome by homologous recombination. The array can then be localized as a green fluorescent protein (GFP)-labeled spot in cells expressing the lac repressor tagged with GFP (Lac I-GFP). Cells within a population are individually analyzed by confocal microscopy and scored as either peripheral, if the Lac I-GFP co-localizes with the nuclear envelope (marked by the endoplasmic reticulum/nuclear envelope membrane protein Sec63-myc), or nucleoplasmic, if the Lac I-GFP does not co-localize with the nuclear envelope [6] (Figure 1A). The *URA3* gene, which distributes randomly within the nucleus, co-localizes with Sec63-myc in 27%–30% of cells [6] (Figure 1A). This represents the baseline for this assay (indicated with a hatched blue line in all relevant figures in this work; [6]). When the *INO1* gene is artificially tethered to the nuclear envelope, we observe peripheral localization in 81% ± 7% of cells [6]. Therefore, the dynamic range of the chromatin localization assay is between 25% and 80%. For this reason, data from chromatin localization experiments were plotted on an axis from 20%–80%. The repressed *INO1* gene distributes randomly, co-localizing with the nuclear envelope in 31% ± 1% of cells in the population (Figure 1A, +inositol; [6]). The activated *INO1* gene is recruited to the nuclear periphery, co-localizing with the nuclear envelope in 60% ± 5% of cells in the population (Figure 1A, −inositol; [6]).

We used the chromatin localization assay to compare the change in the peripheral localization of *INO1* with the change in transcription after shifting cells from repressing to activating conditions. We quantified the levels of *INO1* mRNA relative to *ACT1* mRNA by reverse transcriptase real-time quantitative PCR (RT Q-PCR). After shifting cells into medium lacking inositol, *INO1* mRNA levels increased slowly for the first 2.5 h (Figure 1B, left panel). The mRNA levels then increased more rapidly over the next several hours and reached steady state after 5–6 h (unpublished data). Recruitment of *INO1* to the nuclear periphery was rapid. The fraction of cells in which *INO1* localized to the nuclear periphery increased approximately 10% in the first 5 min after shifting cells to the activating condition and was complete after 60 min. Therefore, *INO1* recruitment to the periphery occurred prior to the rapid accumulation of mRNA. However, plotting the data on a logarithmic scale revealed that there was a substantial fold increase in the concentration of the mRNA during this time, consistent with the possibility that mRNA production might lead to recruitment (Figure 1B, right panel). We conclude that (1) *INO1* was activated quickly, resulting in an approximately 50-fold increase in the mRNA level over the first 45 min, (2) recruitment of *INO1* to the nuclear periphery correlated with this early increase, and (3) the maximal rate of *INO1* mRNA accumulation occurred after relocalization was complete.

We next adapted the chromatin localization assay to compare the localization and transcriptional activation of the *GAL1* gene, which is repressed in cells grown in glucose and expressed in cells grown in galactose. We integrated the lac repressor–binding site array downstream of the *GAL1* gene and quantified its co-localization with the nuclear envelope as in Figure 1A. Repressed *GAL1* localized at the nuclear periphery in 35% ± 1% (five replicates of 30–50 cells) of cells, and activated *GAL1* localized at the nuclear periphery in 70% ± 2.5% (three replicates of 30–50 cells) of cells (unpublished data). When cells were shifted from glucose to galactose, *GAL1* mRNA levels increased slowly for the first 60 min and then more rapidly, reaching steady state after approximately 2 h (Figure 1C). Like *INO1, GAL1* was recruited to the nuclear periphery rapidly, increasing approximately 15% in the first 5 min after shifting cells to galactose medium (Figure 1C). Peripheral localization increased to 56% ± 2% after 60 min (Figure 1C) and continued to increase to 70% over the next 2 h (Figure S1). Therefore, like *INO1,* the rate of accumulation of *GAL1* mRNA was fastest after recruitment to the nuclear periphery.

### Gene Recruitment to the Nuclear Periphery Is Independent of Transcription

**Figure 2:**
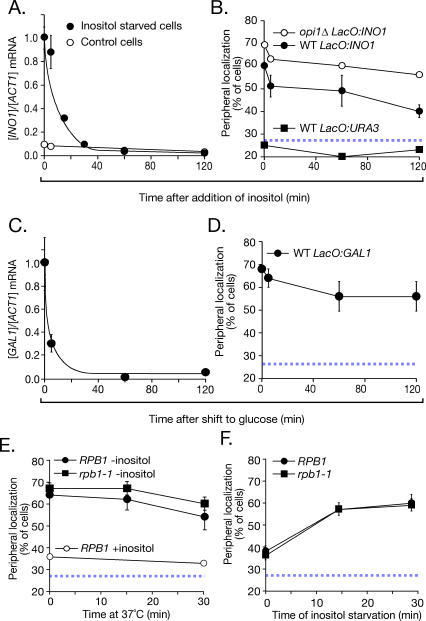
Gene Recruitment Is Maintained after Repression (A and B) The abundance of the *INO1* mRNA ([A], filled circles) and its peripheral localization ([B], filled circles; five replicates of 30–50 cells) in strain JBY397 were quantified at the indicated times after adding 100 μM *myo*-inositol. In (A), a control strain that was grown continuously in the presence of inositol (open circles) was included for comparison. In (B), the localization of *INO1* in the *opi1*Δ mutant (strain JBY404, open circles; two replicates of 30–50 cells), which lacks the repressor of *INO1,* and the localization of *URA3* (strain JBY409, filled squares; one replicate of 50 cells) were included for comparison. WT, wild type. (C and D) Strain DBY32 was shifted from galactose medium to glucose medium, and the abundance of the *GAL1* mRNA (C) and its peripheral localization (two replicates of 30–50 cells (D) were quantified. In (A) and (C), the mRNA ratios were normalized to the initial, fully induced levels. (E and F) Wild-type (strain JBY397) or *rpb1–1* (strain JBY461-r2) cells having the lac repressor array integrated at *INO1* were grown at 25 °C in the absence (E) or presence (F) of inositol. In (E), cells were shifted to 37 °C for the indicated times and scored for peripheral localization (two replicates of 30–50 cells). In (F), cells were first incubated at 37 °C for 15 min before shifting into medium lacking inositol at 37 °C (two replicates of 30–50 cells). Peripheral localization of the *INO1* gene was quantified as in Figure 1. The hatched blue line represents the baseline level of peripheral localization for the *URA3* gene.

We next tested how localization to the nuclear periphery changed after repressing transcription (Figure 2). Both *GAL1* and *INO1* are repressed rapidly [23,24]. After addition of inositol to cells expressing *INO1,* the mRNA levels decreased quickly, with no lag phase, and returned to the fully repressed level within 30 min (Figure 2A). Likewise, in cells shifted from galactose to glucose, the *GAL1* mRNA levels dropped rapidly, with no lag phase (Figure 2C). However, both *INO1* and *GAL1* remained localized at the nuclear periphery for more than 2 h after repressing transcription (Figure 2B and 2D). This persistent localization at the nuclear periphery suggested that these genes are actively retained. The rapid relocalization of both genes upon shifting cells to activating conditions (Figure 1) indicates that they are capable of rapidly changing their distribution. Furthermore, the diffusion coefficient of repressed *GAL1* is approximately 0.18 μm^2^/min [9]. This mobility would predict that, in the absence of an active mechanism of retention, *GAL1* should assume a random distribution within minutes of shifting the cells from activating to repressing conditions.

To rule out the possibility that the localization of *INO1* to the nuclear periphery after repressing transcription was due to very low levels of transcription, we analyzed the localization of *INO1* after inactivating a temperature-sensitive version of RNA polymerase II. RNA polymerase II–mediated transcription is blocked within 5 min after shifting *rpb1–1* mutant cells to the non-permissive temperature (Figure S2 and [25]). We grew *rpb1–1* cells in the absence of inositol to activate *INO1* expression, and then shifted the cells to the non-permissive temperature and quantified the localization of *INO1* to the nuclear periphery over time. After 30 min at the non-permissive temperature, the *INO1* gene remained localized to the nuclear periphery in 60% ± 3% of the cells, despite a 5-fold decrease in *INO1* mRNA levels (Figure 2E and Figure S2A). Therefore, ongoing transcription is not required to maintain *INO1* at the nuclear periphery.

To test if transcription is required to establish *INO1* recruitment, we inactivated RNA polymerase II for 15 min before shifting cells into the activating condition. This treatment completely blocked *INO1* activation, resulting in an approximately 420-fold difference in the levels of *INO1* mRNA (Figure S2B). In the absence of RNA polymerase II function, the *INO1* gene was still recruited rapidly to the nuclear periphery (Figure 2F). These results indicate that transcription is not required for either the establishment or maintenance of gene recruitment to the nuclear periphery. This conclusion is consistent with studies of the interaction of the nucleoporin Nup2 with the *GAL1* promoter [13].

### Gene Recruitment Persists for Generations and Promotes More Rapid Transcriptional Activation

**Figure 3:**
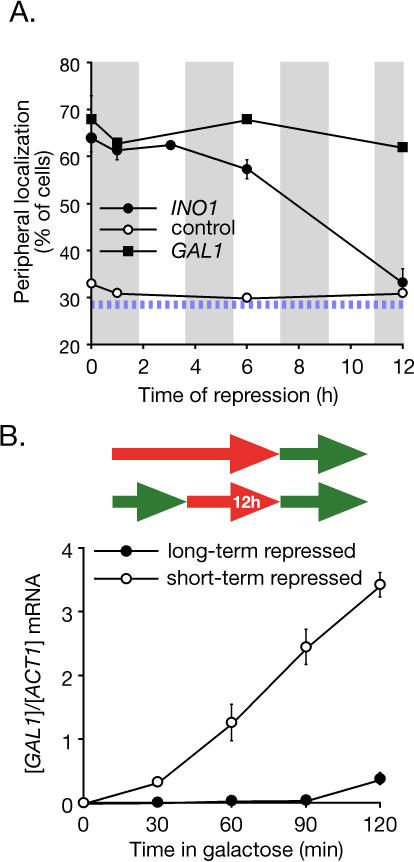
Memory of Recent Transcription. (A) Localization of *INO1* and *GAL1* at the nuclear periphery persists for generations after repressing transcription. Strains JBY397 (lac operator array at *INO1*) and DBY32 (lac operator array at *GAL1*) were shifted from the activating condition to the repressing condition, and the localization at the nuclear periphery was quantified at the indicated times (two replicates of 30–50 cells). Cells were maintained in log phase by continual dilution, and the doubling time was approximately 110 min, indicated as grey vertical bars along the *x*-axis. The hatched blue line represents the baseline level of peripheral localization for the *URA3* gene. (B) Top: schematic of the growth conditions: green arrows indicate growth under activating conditions, red arrows indicate growth under repressing conditions, and inset time indicates the duration of repression. Bottom: *GAL1* activation versus reactivation. Strain BY4741 cultures grown under long-term or short-term (12 h) repressing conditions were shifted into galactose medium. *GAL1* mRNA abundance was quantified at the indicated times using RT Q-PCR and expressed relative to *ACT1* mRNA. Note that strain BY4741 activates *GAL1* more slowly than the CRY1-derived strain used in Figure 1 (Figure S5).

We next tested if the lingering localization of *INO1* and *GAL1* at the nuclear periphery was inherited. We quantified the peripheral localization of both *INO1* and *GAL1* in cells that had repressed transcription through several cell divisions. Cells were maintained in logarithmic growth by continual dilution, and their doubling time was approximately 110 min. The *INO1* gene remained localized at the nuclear periphery in more than 50% of the cells after 6 h of repression and then returned to a random distribution after 12 h (Figure 3A). Therefore, localization of *INO1* at the nuclear periphery was maintained through at least three to four cell divisions. The retention of the *GAL1* gene was even more stable, remaining localized at the nuclear periphery in more than 60% of cells after 12 h of repression (Figure 3A). This suggests that *GAL1* is maintained at the nuclear periphery indefinitely in logarithmically growing cells. Consistent with this indefinite switch, we find that *GAL1* remained localized at the nuclear periphery for greater than 120 h, or approximately 65 generations (Figure S3). Therefore, the localization of *INO1* and *GAL1* at the nuclear periphery is stably maintained after repressing transcription and is inherited by subsequent generations, suggesting that it represents an epigenetic state.

Our data suggest that there are two different forms of repressed *INO1* and *GAL1*. Whereas *INO1* and *GAL1* that have been repressed for many generations distribute randomly within the nucleus, recently repressed *INO1* and *GAL1* localize at the nuclear periphery. Therefore, peripheral localization distinguishes between recently repressed and long-term repressed states. This raised the possibility that localization might function as an epigenetic marker to allow cells to “remember” recent transcription of these genes, potentially affecting their rate of reactivation. To test this idea, we compared the rate of transcriptional activation of long-term repressed and short-term repressed *GAL1*. The rate of reactivation of *GAL1* in cells in which the gene had been repressed for 12 h (six to seven generations) was much more rapid than in cells grown continuously in glucose (Figure 3B). Thus, in a culture in which only approximately 1% of the cells have previously experienced galactose, the reactivation of the *GAL1* gene is enhanced.

**Figure 4:**
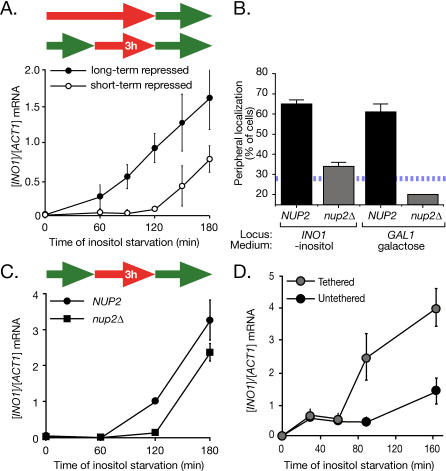
Localization at the Nuclear Periphery Is Necessary and Sufficient to Promote Reactivation of *INO1* (A) *INO1* activation versus reactivation. Strain BY4741 cultures grown under long-term or short-term (3 h) repressing conditions were shifted into medium lacking inositol. *INO1* mRNA abundance was quantified at the indicated times of inositol starvation using RT Q-PCR and expressed relative to *ACT1* mRNA. (B) Wild-type (JBY397 for *INO1,* DBY32 for *GAL1;* four replicates of 30–50 cells) and *nup2*Δ (JBY462 for *INO1,* JBY467 for *GAL1;* two replicates of 30–50 cells) strains having the lac repressor–binding site array integrated at *INO1* or *GAL1* were scored for peripheral localization under activating conditions. The hatched blue line represents the baseline level of peripheral localization for the *URA3* gene. (C) Schematic of the growth conditions: green arrows indicate growth under activating conditions; red arrows indicate growth under repressing conditions. After 3 h of repression with 100 μM inositol, wild-type (CRY1) or *nup2*Δ (JBY451-r1) mutant cells were shifted to medium lacking inositol, and *INO1* mRNA levels were quantified at the indicated times. (D) Tethering of *INO1* to the nuclear periphery enhances the rate of activation. Strains having the lac operator array integrated upstream of the *INO1* gene were transformed with either wild-type Lac I-GFP (JBY397) or Lac I-FFAT-GFP (JBY399) to target the gene to the nuclear membrane [6]. These strains were shifted into medium lacking inositol for the indicated times, and *INO1* mRNA levels were quantified.

We next compared the rate of activation of long-term repressed *INO1* to the rate of reactivation of short-term repressed *INO1* after 3 h of repression (∼1.5 generations). In contrast to *GAL1,* the reactivation of the *INO1* gene after 3 h of repression was delayed compared with activation of the long-term repressed gene (Figure 4A). However, this rate of reactivation was clearly enhanced by the localization at the nuclear periphery. Nup2, a component of the nuclear pore complex that physically associates with transcriptionally active genes such as *GAL1* [7,13], is required for recruitment of both *INO1* and *GAL1* to the nuclear periphery (Figure 4B). Mutants lacking Nup2 exhibited a delay in the reactivation of *INO1* (Figure 4C), suggesting that recruitment to the nuclear periphery promotes more rapid reactivation.

To determine if recruitment to the nuclear periphery is sufficient to promote activation, we compared the activation of *INO1* that was artificially tethered to the nuclear envelope to untethered *INO1*. The lac repressor array was integrated beside *INO1* in strains expressing either the wild-type Lac I-GFP (untethered *INO1*) or a modified version possessing an FFAT motif to target the protein to the nuclear envelope (tethered *INO1;* [6,26]). Expressing this form of the lac repressor results in efficient targeting of the *INO1* gene to the nuclear envelope [6]. Tethering the *INO1* gene to the nuclear envelope had no effect on steady-state levels of *INO1* mRNA under activating or repressing conditions (Figure S4). However, tethered *INO1* was activated more rapidly than untethered *INO1* (Figure 4D). Therefore, localization at the nuclear periphery enhances the rate of reactivation of *INO1*.

If so, then why is the rate of reactivation of recently repressed *INO1* slower than the rate of activation of long-term repressed *INO1*? This difference is likely due to differences in the physiology of cells grown continuously in the presence of inositol and cells to which inositol has recently been added. Activation of *INO1* is regulated by the concentration of phosphatidic acid, a lipid precursor of phosphatidylinositol [27]. Phosphatidic acid consumption is stimulated by both exogenous inositol and the action of the Ino1 enzyme [27]. After repressing *INO1* transcription, the Ino1 enzyme in the cells will continue to produce inositol, driving a higher flux through the pathway and depleting phosphatidic acid. We think this may explain the longer lag phase in the reactivation experiment, which represents the time required for phosphatidic acid to accumulate to levels that activate transcription. This feedback, combined with the shorter duration of the memory phenomenon for the *INO1* gene, complicates a direct comparison between the rate of activation between the short- and long-term repressed states of *INO1*.

### Histone Variant H2A.Z Is Required for Transcriptional Memory

**Figure 5:**
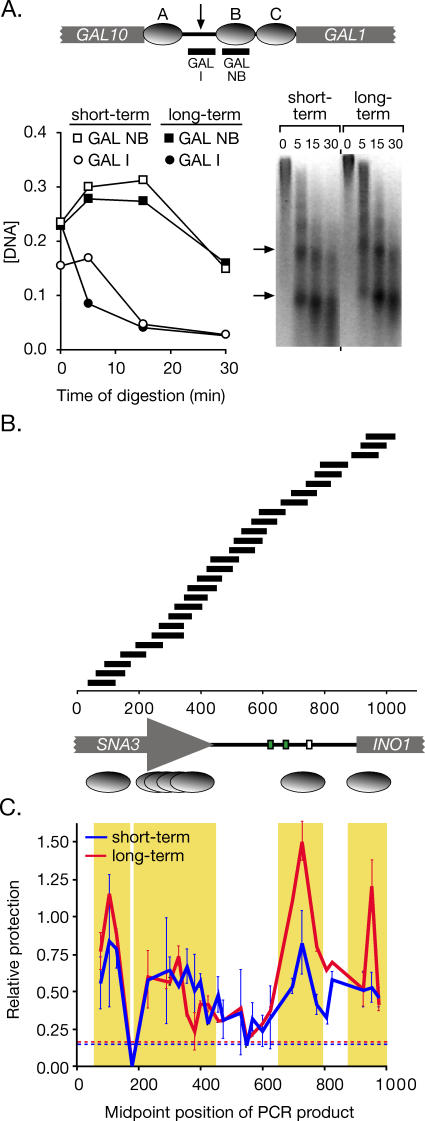
Nucleosome Positioning and Relative Occupancy in the Long-Term Repressed and Short-Term Repressed *INO1* Promoters (A) Top panel: a map of three known, well-positioned nucleosomes, called “A,” “B,” and “C” (grey ovals) within the *GAL1–10* promoter [33]. PCR products to monitor the concentration of sequences protected by nucleosome “B” (GAL NB) or sequences from the inter-nucleosomal region (arrow; GAL I) are indicated below. Bottom panel: DNA from either long-term repressed (open symbols) or short-term repressed (1 h; filled symbols) strain CRY1 was digested with micrococcal nuclease. The concentration of the templates for GAL NB (squares) and GAL I (circles) was determined relative to intact yeast genomic DNA by Q-PCR and plotted against different time points of digestion. Right: inverted image of ethidium bromide–stained gel of the digestion reactions. (B) A map of the *INO1* promoter and flanking sequences, along with the positions of PCR products quantified to analyze nuclease protection and nucleosome occupancy. Green boxes represent UAS_INO_ elements, and the white box represents the TATA box. Below the map, well-positioned nucleosomes are indicated as single ovals, and poorly positioned nucleosomes are indicated as an overlapping series. (C) DNA from short-term and long-term repressed cells digested with micrococcal nuclease for 30 min (A) was analyzed by Q-PCR. Relative protection of the templates for each PCR product in (B) was calculated as a ratio of the concentration of the GAL NB template and mapped using the midpoint of the PCR product. Error bars represent standard error. The hatched lines represent the relative protection of the non-nucleosomal GAL I for each sample. Yellow boxes highlight protected sequences.

To explore the molecular nature of the difference between short-term and long-term repressed *INO1,* we asked if remaining at the nuclear periphery after repression affects the chromatin state of the gene. Because nucleosome remodeling is important for both *INO1* activation and repression [28–32], we compared the positioning of nucleosomes within the short-term repressed and long-term repressed *INO1* promoter. Permeablized cells were treated with micrococcal nuclease for various times to digest unprotected DNA (Materials and Methods). As an internal control for nucleosome protection, we used a known, well-positioned nucleosome within the *GAL1* promoter (GAL NB; Figure 5A; [33]) and an adjacent, non-nucleosomal sequence (GAL I; Figure 5A). Using Q-PCR to define the concentration of these two sequences in our digestion, we observed protection of the nucleosomal sequence relative to the non-nucleosomal sequence (Figure 5A, left panel). Furthermore, after 15 min and 30 min of digestion, we observed the production of clear mononucleosome and dinucleosome bands, indicating that nucleosomes were providing protection from the nuclease and that linker DNA had been digested (Figure 5A, right panel, arrows). Previous studies have established that relative nucleosomal protection is observable over a large range of digestion and with or without gel purification of mononucleosomes [34]. Therefore, we used Q-PCR and a set of 27 different primer pairs to amplify overlapping 80–100 base pair fragments from the *INO1* promoter (Table S1). The concentration of each of the templates for these 27 primer pairs was quantified after 30 min of digestion. The protection of each template was calculated relative to the GAL NB sequence. Using this method, we identified one well-positioned nucleosome within the *INO1* promoter and one at the junction between the promoter and the transcript (Figure 5B). Comparison between short-term and long-term repressed *INO1* revealed no significant change in the positioning of these nucleosomes. However, we did observe a decrease in the relative protection provided by these two nucleosomes in the short-term repressed state (Figure 5C). This difference resulted in a 2-fold decrease in the protection at these two sites relative to the GAL NB site. This difference may reflect either an increase in the fraction of cells in the population in which these nucleosomes are absent, or a change in the stability of these nucleosomes in the lysates subjected to nuclease digestion.

The positioning of the pair of nucleosomes present in the *INO1* promoter suggested that they might contain the histone H2A variant H2A.Z. H2A.Z is incorporated into pairs of nucleosomes that are typically found in the promoters of repressed genes, and incorporation of H2A.Z has been proposed to promote more rapid activation [35–38]. However, genome-wide chromatin immunoprecipitation experiments did not demonstrate a strong association of H2A.Z with the long-term repressed *INO1* promoter [37].

**Figure 6:**
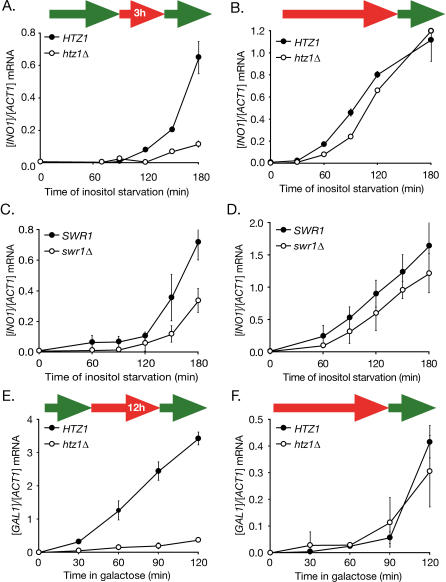
Htz1 Is Required for Transcriptional Memory (A and B) Strains BY4741 and BY4741 *htz1*Δ from either short-term (3 h) repressing conditions (A) or long-term repressing conditions (B) were shifted into medium without inositol and collected at the indicated time points. The *INO1* and *ACT1* mRNA levels were quantified by RT Q-PCR. (C and D) Strains BY4741 and BY4741 *swr1*Δ from either short-term (3 h) repressing conditions (A) or long-term repressing conditions (B) were shifted into medium without inositol and collected at the indicated time points. The *INO1* and *ACT1* mRNA levels were quantified by RT Q-PCR. (E and F) Strains BY4741 and BY4741 *htz1*Δ from either short-term (12 h) repressing conditions (E) or long-term repressing conditions (F) were shifted into galactose medium and collected at the indicated time points. The *GAL1* and *ACT1* mRNA levels were quantified by RT Q-PCR.

Yeast H2A.Z is encoded by the non-essential *HTZ1* gene [39]. To test if H2A.Z is important for transcriptional memory, we compared the rates of reactivation of recently repressed *INO1* and *GAL1* in wild-type and *htz1*Δ mutant cells (Figure 6). Loss of H2A.Z led to a strong delay in the rate of reactivation of both short-term repressed *INO1* and short-term repressed *GAL1* (Figure 6A and 6E). Surprisingly, loss of H2A.Z had no effect on the rate of activation of long-term repressed *INO1* or *GAL1* (Figure 6B and 6F). These results suggest that H2A.Z plays an important and specific role in the reactivation of these genes. H2A.Z is exchanged for H2A within intact nucleosomes by the SWR1 ATPase complex [40–42]. To test if SWR1 plays a role in the H2A.Z-dependent reactivation of *INO1,* we next tested the effect of loss of SWR1 on *INO1* activation and reactivation. We find that *swr1*Δ mutant strains were also defective for reactivation of recently repressed *INO1* (Figure 6C)*,* and had little effect on the activation of long-term repressed *INO1* (Figure 6D).

**Figure 7:**
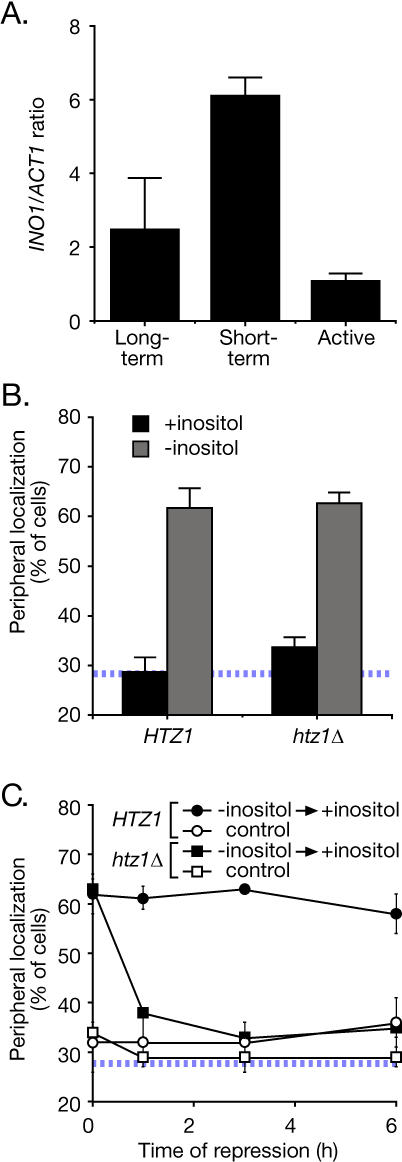
H2A.Z Associates Specifically with the Recently Repressed *INO1* Promoter and Is Specifically Required to Maintain *INO1* at the Nuclear Periphery after Repression (A) Chromatin immunoprecipitation analysis of H2A.Z association with the *INO1* promoter. Strain CRY1 was grown under activating, long-term repressing or short-term repressing (1 h) conditions, fixed with formaldehyde, and processed for immunoprecipitation using anti-Htz1 antibodies (Abcam). Recovered *INO1* promoter was quantified by Q-PCR [6] and expressed relative to recovered *ACT1* coding sequence. (B) H2A.Z is not required for gene recruitment. Wild-type (JBY397) and *htz1*Δ (DBY50) strains were grown either in the presence or absence of inositol and scored for localization at the nuclear periphery. Data are averages of five *(HTZ1)* or three *(htz1*Δ*)* replicates of 30–50 cells. (C) H2A.Z is required for transcriptional memory. Wild-type and *htz1*Δ strains were grown in the absence of inositol to activate *INO1*. Inositol was added to 100 μM and cells were collected for immunofluorescence at the indicated times after repressing transcription. Each time point represents an average of two replicates of 30–50 cells. The hatched line in (B) and (C) represents the baseline level of peripheral localization for the *URA3* gene.

To examine the deposition of H2A.Z nucleosomes in the *INO1* promoter, we used chromatin immunoprecipitation with antiserum against Htz1. Consistent with previous work, immunoprecipitation of H2A.Z from either long-term repressed cells or the activated cells gave low recovery of the *INO1* promoter (Figure 7A). In contrast, immunoprecipitation of H2A.Z from recently repressed cells gave a clear enrichment for the *INO1* promoter (Figure 7A), suggesting that H2A.Z is specifically incorporated into promoter nucleosomes in the recently repressed state.

We next tested if H2A.Z had any role in the localization of the *INO1* gene. Loss of H2A.Z had no effect on recruitment of activated *INO1* to the nuclear periphery (Figure 7B). This is not surprising since the histone variant generally associates with repressed genes (Figure 7A; [35–38]). However, cells lacking H2A.Z were unable to retain *INO1* at the nuclear periphery after repressing transcription (Figure 7C). Therefore, H2A.Z nucleosomes in the recently repressed *INO1* promoter function both to retain recently repressed *INO1* at the nuclear periphery and to promote optimal reactivation.

## Discussion

Our results show that the recruitment of genes to the nuclear periphery is a rapid, active process that is independent of transcription. The most robust transcription of the *GAL1* and *INO1* genes occurred after these genes had fully relocalized to the nuclear periphery, suggesting that recruitment to this subnuclear environment allows optimal expression of these genes. Furthermore, both genes remained at the periphery for generations after repressing transcription, suggesting that cells can inherit localization information. Retention of the *INO1* gene and optimal reactivation of both *INO1* and *GAL1* required the histone variant H2A.Z, which associated with nucleosomes within the recently repressed *INO1* promoter. Thus, cells have both molecular and cellular sources of memory of past transcriptional activation, and they are able to pass on this information to their progeny. This type of memory is mediated by local changes in chromatin structure that mark recently repressed genes to alter their transcriptional potential and localization, and perhaps to provide a mechanism for inheritance.

What is the functional significance of this epigenetic memory? In the case of the *GAL1* gene, the recently repressed state is reactivated much more rapidly than the long-term repressed state, which presumably confers an adaptive advantage upon cells that have previously grown in galactose. We do not see this for *INO1,* perhaps because physiological differences between recently repressed and long-term repressed cells complicates the comparison of the rate of *INO1* activation and reactivation. However, we can conclude that (1) there are two distinct states of repressed *INO1* and *GAL1,* distinguishable by their localization, their transcriptional histories, and the molecular requirements for activation, (2) localization of *INO1* at the nuclear periphery is necessary and sufficient to promote more rapid activation, and (3) incorporation of H2A.Z is the molecular mechanism of transcriptional memory, retaining *INO1* at the nuclear periphery and promoting reactivation of both *INO1* and *GAL1*.

Histone variant H2A.Z is enriched in pairs of nucleosomes within the promoters of repressed genes [35–38]. The histone appears to play an important role in the loss of nucleosomes from promoters upon their activation [37]. This observation, coupled with the fact that H2A.Z nucleosomes are less tightly bound to DNA than H2A nucleosomes, suggests that H2A.Z nucleosomes promote activation by being more easily removed [37]. We find that H2A.Z deposition and function can depend on the transcriptional history of the promoter into which it is incorporated. H2A.Z is required for rapid reactivation of short-term repressed *INO1* and *GAL1* and for retention of recently repressed *INO1* at the nuclear periphery. It is possible that these results represent an indirect effect of loss of H2A.Z. However, we think H2A.Z most likely plays a direct role in promoting the reactivation of *INO1* and *GAL1* because (1) loss of H2A.Z (and SWR1) affects reactivation of recently repressed *INO1* and *GAL1*, but not the activation of long-term repressed *INO1* and *GAL,1* and (2) H2A.Z physically associates with the recently repressed *INO1* promoter. Therefore, we have identified a new and novel role for this histone variant: H2A.Z can serve as a molecular identifier of recently repressed genes to promote their retention at the nuclear periphery and their rapid reactivation.

**Figure 8:**
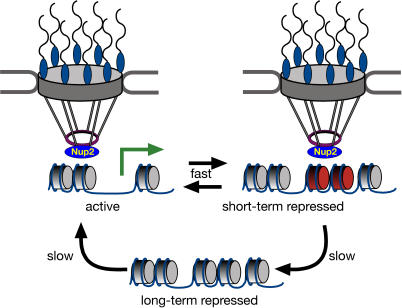
A Model for Transcriptional Memory The long-term repressed state of *INO1* and *GAL1* localizes randomly within the nucleoplasm and is activated slowly. Upon activation, these genes are recruited to the nuclear periphery through interaction with NPC-associated Nup2, with full transcriptional activation following recruitment. Upon repression, *INO1* and *GAL1* remain at the nuclear periphery. Specific incorporation of the histone variant H2A.Z into the recently repressed promoter mediates retention at the nuclear periphery and rapid reactivation.

Our current model for the mechanism of gene recruitment and transcriptional memory is shown in Figure 8. In response to signals that regulate transcriptional activation, genes physically interact with the nuclear pore complex via the mobile nucleoporin Nup2. Recruitment to the nuclear periphery allows access to the optimal subnuclear environment for transcription and, potentially, for mRNA export. After transcription is repressed, previous transcriptional activation of genes such as *INO1* and *GAL1* is remembered through retention in this optimal environment. Localization at the nuclear periphery is epigenetically inherited and requires incorporation of histone variant H2A.Z. Finally, the reactivation of *INO1* and *GAL1* is optimized by both localization at the periphery and through more rapid loss of H2A.Z nucleosomes [37].

What is the role of DNA localization in promoting transcriptional memory? Our data suggest two possible models for how peripheral localization affects H2A.Z-mediated transcriptional memory. In the first model, H2A.Z incorporation into promoter nucleosomes is promoted by Nup2-mediated gene recruitment to the nuclear periphery, and functions to promote reactivation by altering the rate of nucleosome loss or local histone modifications. This model is consistent with several observations in the literature. Tethering of Nup2 to DNA promotes “boundary activity,” insulating euchromatin from the spread of heterochromatin [43,44]. Intriguingly, one of the most dramatic phenotypes of mutants lacking either Nup2 or H2A.Z is the spread of silenced heterochromatin [43,45]. Thus, it is possible that tethering genes to the nuclear periphery through Nup2 leads to the incorporation of H2A.Z nucleosomes, which functions as a boundary. Furthermore, it is possible that boundary elements normally associate with the NPC. We find that H2A.Z is involved in both the activation of recently repressed genes and their retention at the nuclear periphery. Thus, a second model for the importance of H2A.Z is that H2A.Z nucleosomes promote reactivation of recently repressed genes by retaining them in the optimal environment for transcriptional activation. These models are not mutually exclusive, and we favor the possibility that H2A.Z incorporation is promoted by localization and that, once incorporated, H2A.Z affects localization.

Transcriptional memory is employed extensively during development in multi-cellular organisms. In *Drosophila,* Hox gene expression throughout development is determined early in embryogenesis by transcriptional regulators that control segmentation [46]. The initial expression states defined by the segmentation genes are maintained by the action of either polycomb group proteins (generally repressive) or trithorax group proteins (generally activating) through a number of chromatin-based mechanisms such as nucleosome positioning and histone modification [47]. Similarly, the variant histone H3.3 is incorporated selectively into transcriptionally active parts of the genome, which may promote the epigenetic maintenance of an activated state [20,48]. Like these forms of transcriptional memory, the transcriptional memory described here is mediated by chromatin-based changes that mark recently repressed genes and distinguish them from long-term repressed genes. However, unlike these forms of memory, which serve to maintain a previously established transcriptional state, the transcriptional memory described here serves an informational role, revealing previous transcriptional activity and altering the transcriptional potential of previously expressed genes.

Previous work has hinted that transcriptional activity of *GAL1* can alter the degree of methylation of histone H3, marking the chromatin for hours after repressing transcription [19]. However, in this case, the mark was lost after cell division. Our data suggest that the past experiences of microbial organisms can affect their cellular organization and their physiology for many generations. The efficiency of inheritance of the memory state was different for the two genes we examined, suggesting that there are different timing mechanisms for each. In the case of the *GAL1* gene, after exposure to galactose, logarithmically growing cells appeared to undergo an indefinite switch to the recently repressed state. It will be fascinating to determine if there are conditions or stimuli that can reset the *GAL1* gene to the long-term repressed state. In contrast, the transcriptional memory of *INO1* activation was relatively short lived. The previous transcriptional state of *INO1* is imprinted in its chromatin and its subnuclear localization for 6 h or more (two to three cell doublings), but this information is eventually lost.

Why do cells optimize reactivation of genes? We speculate that rapid reactivation of certain genes confers an adaptive, and therefore an evolutionary, advantage to cells. This might be particularly important in the case of stress-responsive genes such as *INO1* or genes involved in metabolizing non-glucose hexose sugars. Also, epigenetic mechanisms may be useful in allowing cells to alter their transcriptional output rapidly under highly variable environmental conditions or under physiological circumstances in which they rapidly undergo reversible changes in physiology [49]. It will be interesting to see if this mechanism is also operative in metazoan organisms, perhaps to establish epigenetically “primed” states for dynamically regulated genes in response to transient physiological or environmental cues.

## Materials and Methods

### Chemicals and reagents.

Unless stated otherwise, chemicals were from Sigma (St. Louis, Missouri, United States), oligonucleotides were from Operon (Huntsville, Alabama, United States), restriction enzymes were from New England Biolabs (Ipswitch, Massachusetts, United States), yeast media components were from Q-Biogene (Irvine, California, United States), antibodies against GFP and myc were from Invitrogen/Molecular Probes (Carlsbad, California, United States), and antiserum against Htz1 was from Abcam (Cambridge, Massachusetts, United States).

### Strains, plasmids, and growth conditions.

Yeast strains used in this study are listed in Table 1. Except for BY4741, BY4741 *htz1*,Δ and BY4741 *swr1*Δ [50], all strains were constructed from CRY1 (*ade2–1 can1–100 his3–11,15 leu2–3,112 trp1–1 ura3–1 MAT* **a**) [51]. Strain JBY451 is the product of a cross between JBY376 (*ade2–1 can1–100 his3–11,15 leu2–3,112 trp1–1 ura3–1 INO1:LacO128:URA3 HIS3:LacI-GFP MAT* **a**) and BY4742 *nup2*Δ mutant strain (*his3*Δ*1 leu2*Δ*0 lys2*Δ*0 ura3*Δ*0 nup2*Δ*::Kan^r MAT*α) from the genome-wide null mutant collection [50]. Random spores JBY451-r1 and JBY451-r7 were selected. For JBY451-r7, the identity of the *ura3* allele was confirmed to be *ura3–1* by transforming JBY451-r7 with StuI-digested pRS306 [52]. Strain JBY462 was created by transforming JBY451-r1 with pRS304-Sec63-myc digested with NheI. Strain JBY467 was created by transforming JBY451-r7 with p6LacO128GAL1 and pRS304-Sec63-myc. Finally, strains JBY451-r1, JBY451-r7, JBY462, and JBY467 were confirmed to be *nup2*Δ by PCR from genomic DNA. Strain JBY461 is the product of a cross between JBY397 [6] and JCY218 [53]. Random spores were selected that were Ura^+^ Trp^+^ His^+^ and temperature sensitive for growth (JBY461-r2). These were then visually scored for expression of Lac I-GFP. Strain DBY50 is the product of a cross between DBY49 (*htz1*Δ*::His5^+^ ade2–1 can1–100 his3–11,15 leu2–3,112 trp1–1 ura3–1 MAT*α) and JBY397. The resulting diploid was sporulated, and tetrads were dissected to generate DBY50.

**Table 1:**
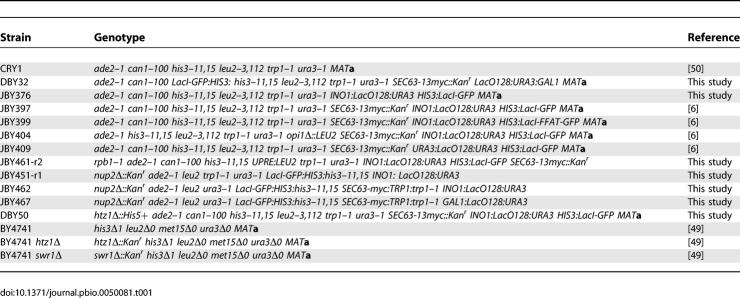
Strains Used in This Study

Plasmids p6LacO128 [6], p6LacO128-INO1 [6], pAFS144 [21], and pAFS144-FFAT [6] have been described. To create the plasmid p6LacO128-GAL1 to mark the *GAL1* gene with the lac repressor–binding site array, the 3′ end of the *GAL1* gene, and downstream sequences were amplified by PCR using the following primers (5′ to 3′): GAL1up, GTTCAAACCGCAGTTGAAGG and GAL1down, CCGAAAGATCTTCTCTATGGGG. The resulting PCR product was cloned into the TOPO4 vector (Invitrogen). This was then cloned into p6LacO128 as a SpeI fragment. The plasmid was integrated downstream of *GAL1* by digestion with NruI.

Plasmid pRS304-Sec63-myc was created by amplifying *SEC63-13myc* from JBY397 genomic DNA using the following primers (5′ to 3′): SEC63up: GTATTTCGGAGAGGGGGC; Pringledown: ACTATACCTGAGAAAGCAACCTGACCTACA. The resulting PCR product was TOPO cloned into pCR2.1 (Invitrogen). The insert was then cloned into pRS304 [52] as a NotI-KpnI fragment. The plasmid was digested with NheI to target integration at *SEC63*.

Unless noted otherwise, all experiments were performed on cells grown in synthetic complete medium at 30 °C. For experiments involving *INO1,* cells were grown in medium lacking inositol or supplemented with 100 μM *myo*-inositol. For experiments involving *GAL1,* cells were grown in media with either 2% glucose or 2% galactose.

### RT-QPCR.

RNA was prepared as described [54]. A total of 2–4 μg of DNAse-treated total RNA was reverse transcribed using 5 μM Oligo dT and 20 units of Superscript III reverse transcriptase (Invitrogen) at 42 °C for 1 h. The reaction was diluted 5-fold, and 1/20th was used for Q-PCR. The sequences of the primers used for real-time PCR were (5′ to 3′): INO1CDS F, TAGTTACCGACAAGTGCACGTACAA; INO1CDS R TAGTCTTGAACAGTGGGCGTTACAT; ACT1CDS F, GGTTATTGATAACGGTTCTGGTATG; ACT1CDS R, ATGATACCTTGGTGTCTTGGTCTAC; GAL1CDS F, GTTCGATTTGCCGTTGGACGG; GAL1CDS R, GGCAAACCTTTCCGGTGCAAG. The relative concentration of cDNA templates for both the target gene *(INO1* or *GAL1)* and the control gene *(ACT1)* were calculated for each sample using standard curves for each primer set that were defined by linear regression analysis of Ct values using a series of 5-fold dilutions of yeast genomic DNA covering a 3,125-fold range.

### Nucleosome scanning.

Long-term repressed cells were harvested at an optical density (OD_600_) of 0.8–1.0 from 1 l of SDC + inositol. Short-term repressed cells were grown in 1 l of SDC − inositol to an OD_600_ of 0.7, and inositol was added to 100 μM. After 1 h of repression, cells were harvested by filtration. Cell permeablization and micrococcal nuclease digestion were performed as described, except that DNA was not size selected [55]. Q-PCR analysis on digested DNA was performed using the oligonucleotides listed in Table S1. To map the protected sequences onto the *INO1* promoter, we used the experimentally determined transcriptional start site and initiation codon [56,57].

### Chromatin immunoprecipitation.

Chromatin immunoprecipitation experiments were performed using anti-Htz1 antiserum (Abcam) as described [37], with the following modifications: 2 μg of anti-Htz1 were used to immunoprecipitate Htz1 from 4.8 mg of chromatin, and immunoprecipitates were recovered using Protein G-dynabeads (Invitrogen). Immunoprecipitated DNA was recovered and analyzed by Q-PCR as described [6]. Recovered *INO1* promoter was expressed relative to recovered *ACT1* coding sequence.

## Supporting Information

Figure S1 Recruitment of *INO1* and *GAL1* to the Nuclear Periphery upon Activation (A) shows an extended time course of the localization of *INO1* shown in Figure 1B, and (B) shows an extended time course of the localization of *GAL1* shown in Figure 1C.
(124 KB TIF)

Figure S2 *rpb1–1* Mutation Blocks RNA Polymerase II Transcription (A) Transcription is blocked in the *rpb1–1* mutant. RNA was isolated from *rpb1–1* strain JBY461-r2 grown in the absence of inositol at 25 °C and then shifted to 37 °C for the indicated times.
(B) *INO1* activation is prevented in the *rpb1–1* mutant. *RPB1* and *rpb1–1* cells were grown in the presence of inositol at 25 °C, shifted to 37 °C for 15 min, and then shifted into medium lacking inositol at 37 °C for 180 min. In both experiments, RNA was reverse transcribed using primers complementary to the 3′ ends of either the *INO1* mRNA (INO1 RT primer: 5′ CAACAATCTCTCTTC) or the RNA polymerase I transcript *RDN18–1* (RDN18–1 RT primer: 5′ CTTAAAATCTCGACC). The resulting cDNA was quantified by Q-PCR using the INO1 CDS primers (Materials and Methods) or RDN18–1 primers (RDN18–1 P1: 5′ TTGTTGCAGTTAAAAAGCTCG and RDN18–1 P2: 5′ TAAAAGTCCTGGTTCGCCAA).
(192 KB TIF)

Figure S3 *GAL1* Remains Localized at the Nuclear Periphery for Days Strain DBY32 was shifted from galactose to glucose medium, and the peripheral localization of the *GAL1* gene was quantified at the indicated times after repression. The hatched blue line indicates the baseline level of peripheral localization of the *URA3* gene.
(58 KB TIF)

Figure S4 Tethering *INO1* to the Nuclear Envelope Has No Effect on Steady-State Levels of *INO1* under Activating or Repressing Conditions Tethered (JBY399) and untethered (JBY397) strains were grown either in the presence or absence of inositol, and the *INO1* mRNA was quantified relative to *ACT1* mRNA by RT Q-PCR.
(82 KB TIF)

Figure S5 *GAL1* Activation in BY4741 and CRY1 Strains CRY1 and BY4741 were grown in glucose medium overnight and shifted to galactose medium. Cells were collected at the indicated times, and *GAL1* and *ACT1* mRNA levels were quantified by RT Q-PCR.
(137 KB TIF)

Table S1 Oligonucleotides Used for Nucleosome Scanning Experiment (55 KB PDF)

## Abbreviations

GFP: green fluorescent protein
NPC: nuclear pore complex
RT Q-PCR: reverse transcriptase real-time quantitative PCR
