# Supplementary material for: H2A.Z-Mediated Localization of Genes at the Nuclear Periphery Confers Epigenetic Memory of Previous Transcriptional State
Source: PLoS Biol. 2007 Mar 20;5(4):e81. doi: 10.1371/journal.pbio.0050081 (PMC1828143; doi:10.1371/journal.pbio.0050081)
Supplement: Supplementary file 1 [file pbio.0050081.st001.pdf]

Table S1. Oligonucleotides used for nucleosome scanning experiment

Name	Forward Primer	Reverse Primer	Position[†]	Midpoint[§]
GAL1 NB	CCCCACAAACCTTCAAATTAACG	CGCTTCGCTGATTAATTACCC	II:278751-278850	N/A
GAL1 I	GTAATACGCTTAACTGCTCATTGC	ACGCACGGAGGAGAGTCTTC	II:278532-278626	N/A
INO1_1	GGACAGAGACCATATTAATGACCAT	GAATGAAAACCGCCAAAACC	X:136690-136599	74.5
INO1_2	CCATCGAATGAGCTATTCCA	TACCCTTACGCTTCCAGACG	X:136663-136569	103
INO1_3	GGACGACTTGTTGTTAATGGTTTTG	AGTGTATCCCTGTTGAACATACCCT	X:136636-136550	126
INO1_5	TGGAAGCGTAAGGGTATGTTC	GTGAATGATTGCTGGGAAGA	X:136584-136501	176.5
INO1_6	TCTTCCTACTGTTATTCTTCCCAGC	TGCGTGAAAGATCGTACGAA	X:136535-136446	228.5
INO1_8	TGAAACGAGTAGTGAACGTTCGTAC	TGTGCTTGAGATTCCTCTGCA	X:136483-136379	288
INO1_9	ACGATCTTTCACGCAGACAT	TGTGCTTGAGATTCCTCTGCA	X:136460-136379	299.5
INO1_10	CGCCGTAGACCGTGACCT	CATCGTCTTCATCATATGCTGG	X:136429-136353	328
INO1_11	AAGCTCACCCTGCAGAGGAAT	TTGTTGTCCATCAAGGGCACAT	X:136409-136319	355
INO1_12	AGCCTCCAGCATATGATGAA	CGGAAGAGAGCTGTTGTTTGTT	X:136379-136302	378.5
INO1_13	TATGATGAAGACGATGAGGCCG	AAGAGCTCGTTCCGATCACTAA	X:136368-136273	398.5
INO1_14	TGCCCTTGATGGACAACAAA	TTTAGAACTACGGTGATAAAGAGCT	X:136337-136255	423
INO1_15	TTCCGGCCGTACTTAGTGAT	CGAAGAAAAAGGCAATAATTTACTC	X:136306-136221	455.5
INO1_16	TAGTGATCGGAACGAGCTCTTT	GACGTGAAGAACAAAAGGAACG	X:136293-136201	472
INO1_17	GCCTTTTTCTTCGTTCCTTTTG	ACTTTCGATTCCGCATCCAA	X:136233-136149	528
INO1_18	CCTTTTGTTCTTCACGTCCTTTTTA	GCCTCCGCATATTTCACATTC	X:136218-136125	547.5
INO1_19	ATGAAATACGTGCCGGTGTT	GAAGCGCATACTTGGCCT	X:136194-136111	566.5
INO1_20	CGGAATCGAAAGTGTTGAATG	GACAATACTTTTCACATGCCGC	X:136161-136078	599.5
INO1_21	AATATGCGGAGGCCAAGTAT	TCTGGGAAAGAAGGATGAAGA	X:136137-136050	625.5
INO1_25	TCATCCTTCTTTCCCAGAATATTG	CTCAAATTAACATTGCCGCC	X:136067-135979	696
INO1_26	TAATTCACATGGAGCAGAGAAAG	AAAGGTGACGAAAGCTCCAA	X:136035-135948	727.5
INO1_28	ATTTGAGACGTATATAAATTGGAGC	GGATAAAACTAACATTAGGAACCCG	X:135985-135904	774.5
INO1_29	CGTCACCTTTTTTTGGCTTGT	CTGGAAAAAAAAGGGAATGAAAC	X:135957-135869	806
INO1_30	TTGTTCTGTTGTCGGGTTCCTA	GTCATTGTTACTTCTTTTTCACTGG	X:135940-135848	825
INO1_33	TATTGCTCCAATCACCTCCGTT	CATTTTCGTAGCTGTACTTGGTGAG	X:135838-135750	925
INO1_34	TTACCGACAAGTGCACGTACAA	GCCACTAGCTGTCTTCGTAACTACA	X:135809-135724	952.5
INO1_35	TACAAGGACAACGAGCTGCT	TCTTGAACAGTGGGCGTTACAT	X:135792-135695	975.5

[†] (Chromosome: bp-bp) from the Saccharomyces Genome Database (http://www.yeastgenome.org/)

[§] Map position plotted in Figure 6.
